# Identification of cucumber S-adenosylmethionine decarboxylase genes and functional analysis of *CsSAMDC3* in salt tolerance

**DOI:** 10.3389/fpls.2023.1076153

**Published:** 2023-04-21

**Authors:** Mengliang Zhu, Guangling Chen, Jianqing Wu, Jian Wang, Yu Wang, Shirong Guo, Sheng Shu

**Affiliations:** ^1^ Key Laboratory of Southern Vegetable Crop Genetic Improvement, Ministry of Agriculture, College of Horticulture, Nanjing Agricultural University, Nanjing, China; ^2^ Suqian Academy of Protected Horticulture, Nanjing Agricultural University, Suqian, China

**Keywords:** antioxidant metabolism, cucumber, *CsSAMDC3*, polyamines, salt stress

## Abstract

As one of the key enzymes in the biosynthesis of polyamines, S-adenosylmethionine decarboxylase (SAMDC) plays an important role in plant stress resistance. In this study, four *SAMDC* genes *(CsSAMDC1-4)* were identified in cucumber (*Cucumis sativus* L.) and divided into three groups (I, II, and III) by phylogenetic analysis. Motif analysis suggested the existence of many conserved motifs, which is compatible with SAMDC protein classification. Gene structure analysis revealed that *CsSAMDC2* and *CsSAMDC3* in group I have no intron, which showed a similar response to salt stress by gene expression analysis. *CsSAMDC3* responded differently to hormone and stress treatments, and was more susceptible to salt stress. Compared with wild-type (WT) tobacco, the activities of superoxide dismutase, peroxidase, and catalase were increased in *CsSAMDC3*-overexpressing tobacco under salt stress, but the content of electrolyte leakage, malondialdehyde, and hydrogen peroxide were decreased, which alleviated the inhibition of growth induced by salt stress. Under salt stress, overexpression of *CsSAMDC3* in transgenic tobacco plants exhibited salt tolerance, mainly in the form of a significant increase in dry and fresh weight, the maximal quantum yield of PSII photochemistry, the net photosynthetic rate and the content of spermidine and spermine, while the content of putrescine was reduced. In addition, the expression levels of antioxidase-related coding genes (*NtSOD*, *NtPOD*, *NtCAT*) and PAs metabolism-related coding genes (*NtSAMS*, *NtSPDS*, *NtSPMS*, *NtPAO*) in transgentic plants was lower than WT under salt stress, which suggested that overexpression of *CsSAMDC3* affected the expression of these genes. In summary, our results showed that *CsSAMDC3* could be used as a potential candidate gene to improve salt tolerance of cucumber by regulating polyamine and antioxidant metabolism.

## Introduction

Polyamines (PAs) are a class of low molecular weight aliphatic nitrogen compounds with strong biological activity produced during biological metabolism (Kuznetsov and Shevyakova, 2010). The main PAs in higher plants include putrescine (Put), spermidine (Spd), and spermine (Spm), which are widely involved in many aspects of plant growth and development. It includes root elongation (Tang and Newton, 2005), flower development and fruit ripening (Liu et al., 2006), leaf senescence (Mattoo and Sobieszczuk-Nowicka, 2019), cell division and differentiation (Masson et al., 2017), programmed cell death (Moschou and Roubelakis-Angelakis, 2014), transcription and translation (Tiburcio et al., 2014), DNA synthesis (Mustafavi et al., 2018), etc. In recent years, with the in-depth study of PAs, it has been found that there is an extraordinarily complex relationship between PAs and abiotic stress. On the one hand, PAs have cationic properties, they can bind directly to membrane phospholipids, proteins and nucleic acids to maintain functional stability under abiotic stresses (Mbarki et al., 2018). On the other hand, under abiotic stress, PAs can interact with abscisic acid (ABA) and nitric oxide (NO) to activate ion channels (Wimalasekera et al., 2011; Pottosin et al., 2012), as well as regulate stomatal and programmed cell death through signaling molecules such as hydrogen peroxide (H_2_O_2_) (Klingler et al., 2010; Tisi et al., 2011; Moschou et al., 2012), or modulate the expression of nucleoside diphosphate kinase to alter activities of antioxidant enzymes (Khare et al., 2018). In summary, PAs can directly or indirectly maintain cell ion balance, reactive oxygen species (ROS) stability, osmotic pressure balance, etc., and ultimately lead to enhanced plant abiotic stress tolerance.

S-adenosylmethionine decarboxylase (SAMDC) catalyzes the decarboxylation of S-adenosylmethionine (SAM) to produce decarboxylative S-adenosylmethionine (dcSAM), which provides the aminopropyl required for the synthesis of Spd and Spm from Put. It is one of the critical enzymes in the PAs biosynthesis pathway (Slocum et al., 1984). Mellidou et al. (2016) reported that tobacco plants with downregulated SAMDC exhibit reduced PAs synthesis and stress tolerance. Plants treated with methylglyoxal bis (guanylhydrazone), a SAMDC inhibitor, resulted in a reduction in the maximal quantum yield of PSII photochemistry (Fv/Fm) and effective PSII quantum yield (Y (II)), together with higher levels of lipid peroxidation and salt stress damage (Ikbal et al., 2014). In addition, many studies have found that overexpression of *SAMDC* could increase plant biomass and enhance plant resistance to extreme environmental stresses (Li and Chen, 2000; Zhao et al., 2010; Jiao et al., 2022). Under abiotic stress, upregulation of SAMDC can improve antioxidant protective enzyme activitives to protect plant cells from oxidative damage by scavenging ROS (Meng et al., 2021). Thu-Hang et al. (2002) demonstrated that heterologous expression of *SAMDC* in plants could increase the enzyme activity of SAMDC and lead to a significant accumulation of Spd and Spm in rice leaves. Overexpression of *BvSAMDC* in sugar beet *Bv*M14 increases plant salt tolerance by enhancing antioxidant enzymes and reducing ROS production (Ji et al., 2019). Luo et al. (2017) showed that overexpression of *CdSAMDC1* increased the synthesis of Spd and Spm in transgenic centipedegrass, causing an increase in polyamine oxidase activity to generate H_2_O_2_; elevated H_2_O_2_ increased nitrate reductase activity to produce NO, which in turn increased antioxidant enzyme activity and cold tolerance of transgenic plants. Therefore, the role of *SAMDC* under abiotic stress is complex and essential.

So far, genes encoding SAMDC have been identified in many plants, such as wheat (Zeng et al., 2011), soybean (Tian et al., 2004), sugarcane (Liu et al., 2010), navel orange (Wang et al., 2010), carnation flower (Lee et al., 1997), potato (Kumar et al., 1996), tomato (Kolotilin et al., 2011) and upland cotton (Tang et al., 2021). These studies have shown that SAMDC may be encoded by one or more *SAMDC* genes in different species and have different catalytic activity and expression characteristics. However, the in-depth study of cucumber *SAMDCs* has not been reported. In this study, we identified four cucumber *SAMDC* gene family members in the cucumber genome, named *CsSAMDC1*, *CsSAMDC2*, *CsSAMDC3* and *CsSAMDC4*. Firstly, we analyzed the basic sequence information, structure, and sequence homology of these genes and detected the expression pattern of *CsSAMDC1-4* in roots and leaves under salt stress. Secondly, the subcellular localization of *CsSAMDC3* was observed, and its expression patterns in different tissues or under stress were detected. Finally, we further studied the role of *CsSAMDC3* in salt stress response by overexpressing *CsSAMDC3* in tobacco.

## Materials and methods

### Plant materials and stress treatment

Cucumber (*Cucumis sativus* L. cv. Jinyou No. 4) was used as experimental material. The seedlings were cultivated in half-strength Hoagland nutrient solution (pH 6.3 ± 0.2, EC=2.2 ± 0.2 mS·cm^-1^) and cultivated in the artificial climate chamber. The growth temperature was 25°C/16°C (day/night). The photoperiod was 14 h/10 h (day/night) with light intensity at 600 μmol·m^-2^·s^-1^, and relative humidity was stabilized at 60-75%. To analyze the effects of hormones on cucumber seedlings, 100 μM abscisic acid (ABA), 100 μM salicylic acid (SA), 100 μM ethylene (ETH), and 100 μM methyl jasmonate (MeJA) were sprayed on the leaves when the seedlings had two true leaves. To analyze the effects of abiotic stress on cucumber seedlings, 75 mM NaCl and 20% (w/v) polyethylene glycol (PEG) 6000 were added to the Hoagland nutrient solution of cultivated seedlings to simulate salt stress and drought stress, respectively. The seedlings were placed in a 4°C light incubator to simulate cold stress. Leaves and roots were collected at 0, 3, 6, 9, 12, 24, 48, and 72 h after treatment and stored at -80°C for use. To analyze the tissue-specific expression pattern, different tissues of the cucumber reproductive growth period were stored at -80°C.

Wild-type tobacco (*Nicotiana tabacum* L.) and transgenic tobacco (*CsSAMDC3*-overexpressing) were cultured in an artificial climate chamber. When the tobacco grew to four ture leaves, irrigated with 200 mM NaCl. After 5 days of treatment, the leaves and roots were stored at -80°C.

### Identification and sequence analysis of cucumber SAMDC

The amino acid sequence of SAMDC was retrieved from the reported Cucurbit Genomics Database (http://cucurbitgenomics.org/) and verified in NCBI Database. The isoelectric point (pI) and molecular weight (MW) of the candidate SAMDC were calculated using ExPASy (https://www.expasy.org/). To study the evolutionary relationship of SAMDC between cucumber and other plant species, the *SAMDC* gene sequence and SAMDC protein sequence of 12 plants such as pumpkin, tobacco, and soybean were obtained by searching NCBI Genome Database. The SAMDC protein sequences of different species were compared by Clustal W, and the phylogenetic tree was constructed by NJ (neighbor-joining) method, Poisson correction, and 1000 bootstrap replicates in MEGA 11 software. The conserved motifs of different species and the conserved domains of SAMDC protein sequences were searched by the online MEME tool (https://meme-suite.org/meme/) and NCBI CDD (https://www.ncbi.nlm.nih.gov/cdd/), and visualized by TB Tools (Chen et al., 2020).

### Genetic transformation and treatment of tobacco

To construct *CsSAMDC3*-overexpressing tobacco, the primer pair PAC019-*CsSAMDC3*-F/R (**Supplemental Table 1**) was designed. *CsSAMDC3* was cloned from cucumber cDNA with PrimeSTAR^®^ Max DNA Polymerase (Takara, China) following the instructions. The cucumber *SAMDC3* gene fragment was ligated to the digested PAC019 vector using ClonExpress^®^ II One Step Cloning Kit (Vazyme, China). The normal sequencing plasmid was transferred into *Agrobacterium* EHA105 by heat shock method. *CsSAMDC3* was transformed into *Nicotiana tabacum* by *Agrobacterium*-mediated according to the previous research method (Wang et al., 2017). Homozygous transgenic plants were screened by kanamycin and identified by PCR analysis. Quantitative real-time PCR (qRT-PCR) analysis was also performed to validate the transformation of *CsSAMDC3* further. T3 homozygous transgenic lines for further study.

### RNA extraction, reverse transcription, and quantitative real-time PCR

RNA simple Total RNA Kit (Tiangen, China) was used to extract total RNA from samples. HiScript^®^ III Q RT Super-Mix for qPCR Kit (Vazyme, China) reverse transcribed 1μg total RNA into cDNA for gene cloning and qPCR. Based on the selected gene sequence, the primer pair (**Supplemental Table 1**) was designed using Beacon Designer™ 8.10 (Premier Biosoft International, USA). qRT-PCR was conducted on Quant-Studio™ 5 Real-Time PCR System (Applied Biosystems) with ChamQ SYBR qPCR Master Mix (Vazyme, China), which included 10 μL of ChamQ SYBR qPCR Master Mix (2 ×), 0.4 μL of sense or anti-sense primer, 0.4 μL of ROX Reference dye (50 ×), 1 μL of cDNA and 7.8 μL of ddH_2_O in a total volume of 20 μL. The PCR program was as follows: 95°C for 30 s; 40 cycles of 95°C for 10 s, 58°C for 10 s; and finally, 72°C for 30 s.

The relative expression levels of the selected genes were calculated by 2^-ΔΔCT^ method using cucumber or tobacco actin genes as internal controls (Livak and Schmittgen, 2001).

### Electrolyte leakage, malondialdehyde, and hydrogen peroxide determination

The electrolyte leakage rate was measured according to the description of He et al. (2019). 0.5 g fresh sample was immersed in a test tube containing 20 mL deionized water. After shaken at room temperature for 4~5 h, the initial conductivity (EC_1_) was measured. Then, the sample was boiled at 95 °C for 20 min and cooled to room temperature, and the final conductivity (EC_2_) was measured in the bath. We also measured the conductivity of deionized water (EC_0_). EL (%) was calculated as [(EC_1_-EC_0_)/(EC_2_-EC_0_)] × 100.

MDA content was determined according to the description of Dhindsa et al. (1981). The leaves were ground on ice with 5% TCA and centrifuged at 4000 g for 10 min. Then 2 mL of the supernatant was mixed with an equal amount of 0.67% thiobarbituric acid (TBA). After being heated in a boiling water bath for 30 min, the mixture was centrifuged at 3000 g for 15 min. The absorbance of the supernatant was read at 450, 532, and 600 nm and calculated. The MDA content is represented as nmol·g^-1^ FW.

The content of H_2_O_2_ was determined according to the description of Zhu et al. (2021). The 0.2 g leaf sample was ground on ice using 1.6 mL of pre-cooled 0.1% trichloroacetic acid (TCA) to a slurry and then centrifuged at 12000 g for 20 min. The 0.2 mL supernatant was mixed with 1 mL 1 M KI solution and 0.25 mL 0.1 M potassium phosphate buffer (pH 7.8) and placed in the dark for 1 h. The absorbance at 390 nm was read with 0.1% TCA as blank. The content of H_2_O_2_ in the sample was calculated according to the standard curve of known H_2_O_2_ concentration. The H_2_O_2_ content is expressed as μmol·g^-1^ FW.

### Assay of antioxidant enzyme activity

The leaves were ground into a slurry in 50 mM precooled phosphate buffer (pH 7.8), transferred into a 2 mL centrifuge tube, and centrifuged at 4°C, 12000 g for 20 min. The resulting supernatant was the crude enzyme solution for determining antioxidant enzyme activity.

Superoxide dismutase (SOD) activity was determined with reference to the description of Giannopolitis and Ries (1977) with minor modifications. The NBT photoreduction method was used. The reaction mixture contained 50 mM PBS (pH 7.8), 30 μM EDTA-Na_2_, 14.5 mM methionine, 60 μM riboflavin and 2.25 mM nitroblue tetrazolium chloride (NBT). 3 mL reaction mixture was mixed with 40 μL crude enzyme solution, and the tubes were illuminated for 20 min. The absorbance was read at 560 nm. One unit of SOD activity was defined as the amount of enzyme required to cause 50% inhibition of NBT photoreduction.

Peroxidase (POD) and catalase (CAT) were determined according to the description of Wu et al. (2022). The enzyme extract was mixed with the reaction solution. The reaction solution to determine POD was 20 mM phosphate buffer (pH 6.0), 3.5 M guaiacol, and 30% H_2_O_2_. The reaction solution to determine CAT was 50 mM phosphate buffer (pH 7.0) and 30% H_2_O_2_. The absorbance changes at 470 or 240 nm within 40 s were measured. A unit of POD or CAT activity was expressed as a change of OD_470_ or OD_240_ value of 0.01 min^-1^.

### Net photosynthetic rate and chlorophyll fluorescence parameters

The net photosynthetic rate (Pn) of tobacco leaves at the same leaf position was measured by portable photosynthesis system (Li-6400 XT, Li-COR, Lincoln, NE, USA). The light intensity, leaf temperature, relative humidity, and ambient CO_2_ concentration maintained at 800 μmol·m^-2^·s^-1^, 25°C, 70%, and 400 ± 10 μmol·mol^-1^, respectively. Chlorophyll fluorescence was measured by fluorescence imaging system (IMAGING-PAM, Heinz Walz, Effeltrich, Germany). The measured data and collected fluorescence images were analyzed by ImagingWin software (Heinz Walz, Effeltrich, Germany). The chlorophyll fluorescence parameters were measured according to the method of Shu et al. (2016), and the maximal quantum yield of PSII photochemistry (Fv/Fm) was calculated.

### Measurement of PAs content

PAs were measured according to the description of Shu et al. (2012). The sample was ground into a slurry in 1.6 mL 5% precooled perchloric acid (PCA) and then centrifuged at 12000 g at 4°C for 20 min. The supernatant was collected to measure free and bound PAs, while the particles were used to measure bound PAs.

For free PAs, 0.7 mL of supernatant, 1.4 mL of 2 M NaOH, and 15 μL of benzoyl chloride were mixed, then vortexed for 20 s and kept at 30 °C for 30 min. Then, 2 mL saturated NaCl solution, and cold colddiehtyl ether were added to the mixed solution to extract benzoyl polyamines. After centrifugation at 4 °C for 12000 g for 5 min, 1 mL of ether phase was evaporated to dryness and redissolved in 1 mL of 64% (v/v) methanol.

For conjugated PAs, 0.7 mL of supernatant was mixed with 5 mL of 6 M HCl and sealed in an ampoule at 110 °C for 18 h to convert conjugated PAs into free PAs. The hydrolysate was evaporated at 70°C, and the resulting residue was re-suspended in 1.6 mL of 5% PCA. The following steps are the same as free PAs extraction.

For bound PAs, the particles were washed four times with 5% PCA and centrifuged at 3000 g for 5 min. The resulting particles were suspended in 5 mL 6 M HCl and then subjected to the same steps as conjugated PAs extraction.

Samples redissolved in methanol are stored at -20°C and filtered with a membrane (0.45 μm) before testing. UPLC system (Thermo, UltiMate 3000), including ACQUITY UPLC HSS T_3_ column, acetonitrile, and water (volume ratio of 44: 56) as the solvent, the flow rate of 0.45 mL min^-1^ for the detection of PAs content. The sum of three forms of PAs is the total amount of endogenous PAs.

### Subcellular localization analysis

Subcellular localization prediction of CsSAMDC3 using the CELLO tool (http://cello.life.nctu.edu.tw/).

The agrobacterium strain EHA105 transformed with PAC019-*CsSAMDC3* plasmid was transiently transformed into *Nicotiana tabacum* by injection infection method. A confocal laser scanning microscope (LSM 780, Zeiss, Germany) was used for imaging after incubation for 36-48 h under the light.

### Statistical analysis

All data were analyzed by single factor analysis of variance (ANOVA) using IBM SPSS 26.0 software (SPSS Inc., Chicago, IL, USA). Duncan’s multiple comparison method was used to analyze the difference between different treatments at a *P* < 0.05 level of significance.

## Result

### Bioinformatics analysis of *SAMDC* family genes in cucumber

Four *CsSAMDC* gene sequences were identified by PCR amplification and sequencing, which were consistent with the search results of the database. As shown in **Table 1**, *CsSAMDC1* and *CsSAMDC3* are located on chromosome 3, while *CsSAMDC2* and *CsSAMDC4* are located on chromosome 6 and 2, respectively. The length of the *CsSAMDC1-4* gene sequence is relatively close, all of which are about 1100 bp, the encoding amino acid (AA) number is 340-389, the encoding protein isoelectric point (pI) is 4.56-6.88, molecular weight is about 40 KDa, hydrophilic average coefficient (GRAVY) is close to 0, and it is inferred to be amphoteric protein.

**Table 1:** Description of *SAMDC* genes in cucumber.

Gene	Gene ID	Location in chromosome	CDS length (bp)	AA	pI	MW (KDa)	GRAVY
*CsSAMDC1*	CsaV3_3G020560	3	1095	364	4.56	40.1	-0.096
*CsSAMDC2*	CsaV3_6G005510	6	1047	348	6.88	38.8	-0.106
*CsSAMDC3*	CsaV3_3G013880	3	1023	340	6.06	37.8	-0.019
*CsSAMDC4*	CsaV3_2G007750	2	1170	389	4.95	43.0	-0.082

CDS, coding sequence; bp, base pairs; AA, number of amino acids; pI, theoretical isoelectric point; MW, molecular weight; GRAVY, Grand average of hydropathicity.

**Figure 1:**
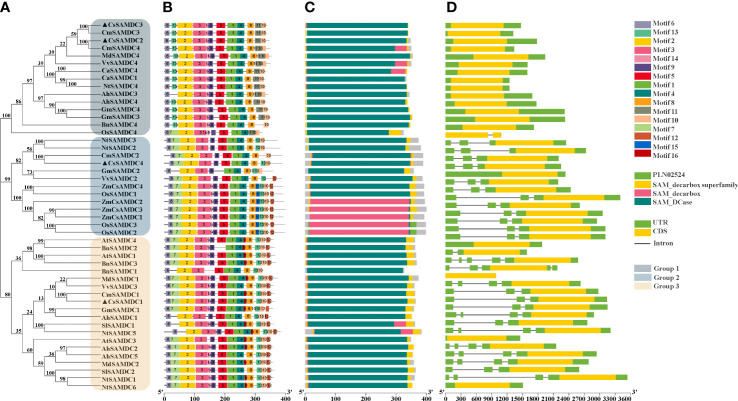
Bioinformatics analysis of SAMDC proteins and *SAMDC* genes from different plants. **(A)** Evolutionary relationships of SAMDC proteins. **(B)** Distribution of the 16 conserved motifs in the SAMDC proteins. **(C)** Protein domains of SAMDC proteins. **(D)** Gene structure of *SAMDC* genes. The accession numbers of SAMDC proteins and *SAMDC* genes used to construct the phylogenetic tree are listed in **Supplemental Table 2**. Motifs are colored by different boxes, and their sequences are listed in **Supplemental Table 3**.

Phylogenetic analysis of the full-length SAMDC protein sequences of 13 species, including cucumber, showed that these SAMDC proteins could be divided into 3 groups, of which CsSAMDC2 and CsSAMDC3 were in group 1, while CsSAMDC4 and CsSAMDC1 were in group 2 and group 3, respectively (**Figure 1A**). Conserved motif analysis of SAMDC protein sequence by the online MEME tool identified 16 conserved motifs. The distribution was consistent with the phylogenetic tree (**Figure 1B**). The conserved domain of the SAMDC protein sequence was analyzed by NCBI CDD. A typical SAMDC protease domain was found (**Figure 1C**). By searching NCBI Genome Database, the entire sequence of the *SAMDC* genes was obtained for visual analysis. The results showed that the *SAMDC* genes of group 1 basically did not contain introns, while the introns of group 2 and group 3 had different lengths (**Figure 1D**). In addition, the CDS regions of most *SAMDC* genes are continuous (**Figure 1D**).

### Response of *SAMDC* family genes to salt stress in cucumber

**Figure 2:**
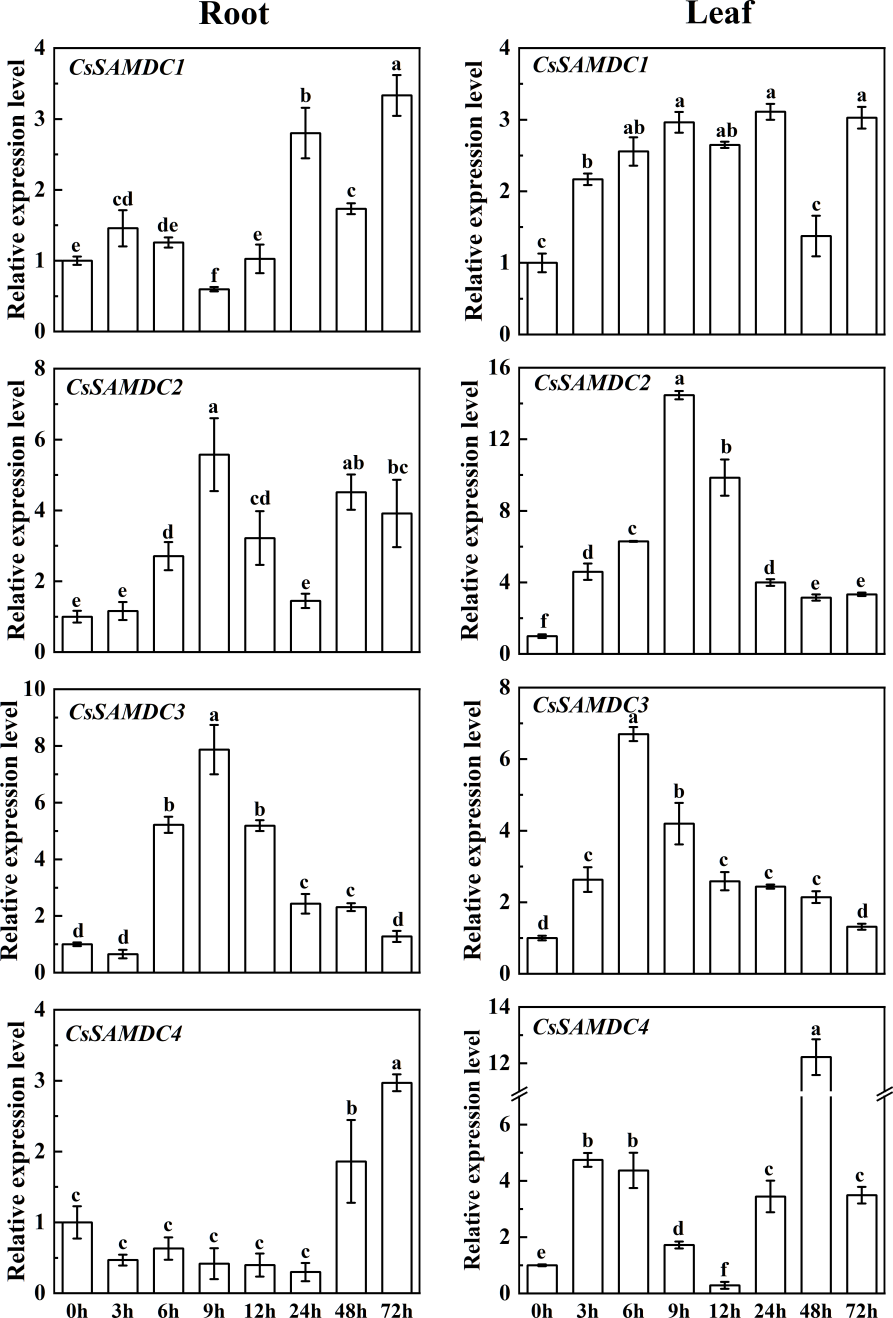
Responses of cucumber *SAMDCs* to salt stressEach value is shown as mean ± standard error of three biological replicates. Different letters indicate significant differences at *P* < 0.05, according to Duncan’s multiple range tests.

The response of cucumber *SAMDC* family genes to salt stress was analyzed by qRT-PCR. As shown in **Figure 2**, there were differences in the expression patterns of *SAMDC* family genes in cucumber roots and leaves. The expression trends of *CsSAMDC2* and *CsSAMDC3* are similar, both of which firstly increased and then decreased. The expression levels of *CsSAMDC2* and *CsSAMDC3* in roots peaked after 9 h of salt treatment, which were 6 times and 8 times higher than that of the control, respectively. The expression of *CsSAMDC2* in leaves also peaked at 9 h, which was 15 times higher than that of the control, while the expression of *CsSAMDC3* peaked earlier at 6 h, which was 7 times higher than that of the control. It suggests that *CsSAMDC3* is the most sensitive to salt stress and the first to respond to salt stress. In addition, the response of *CsSAMDC1* to salt stress was relatively stable in leaves, while its expression level in roots was close to that of the control within 24 h of salt stress and then increased. The response of *CsSAMDC4* to salt stress was more prominent in leaves, and its expression peaked at 48 h, which was 12 times higher than that of the control, while its expression in roots showed a trend of slight decrease and then increase. After that, we selected *CsSAMDC3*, which is sensitive to salt stress, as a typical case for further study.

### Tissue-specific expression and subcellular localization of *CsSAMDC3*

**Figure 3:**
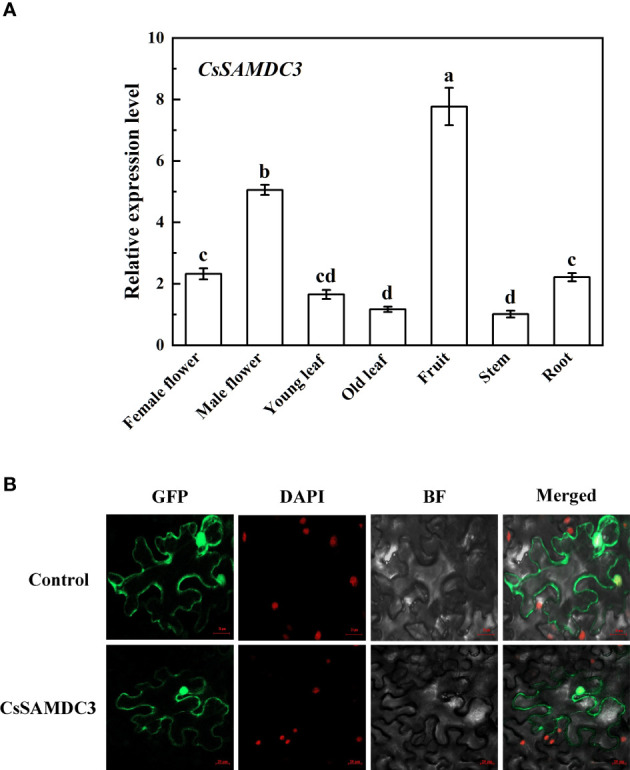
Tissue-specific expression of *CsSAMDC3* **(A)** and subcellular localization **(B)** in cucumber. Expression level in stem is taken as 1. Each value is shown as mean ± standard error of three biological replicates. Different letters indicate significant differences at *P* < 0.05, according to Duncan’s multiple range tests.

*CsSAMDC3* was expressed in different tissues of cucumber. The expression level of *CsSAMDC3* in fruit and flower was 5-8 times higher than that in leaves. In comparison, the expression level in the root, stem, and leaf was lower (**Figure 3A**), indicating that *CsSAMDC3* was mainly related to flower organ development.

Subcellular localization prediction of CsSAMDC3 showed that the protein was likely to be localized in the cell membrane and nucleus (**Supplemental Figure 1**). To verify the results of CELLO subcellular localization prediction, the green fluorescence signal of GFP-CsSAMDC3 fusion protein was detected by ultra-high resolution laser confocal microscopy. **Figure 3B** showed that the green fluorescence signal of the fusion protein was on the cell membrane and nucleus, indicating that CsSAMDC3 was located on the cell membrane and nucleus. The results were consistent with CELLO prediction.

### Response of *CsSAMDC3* to different hormone and abiotic stresses

**Figure 4:**
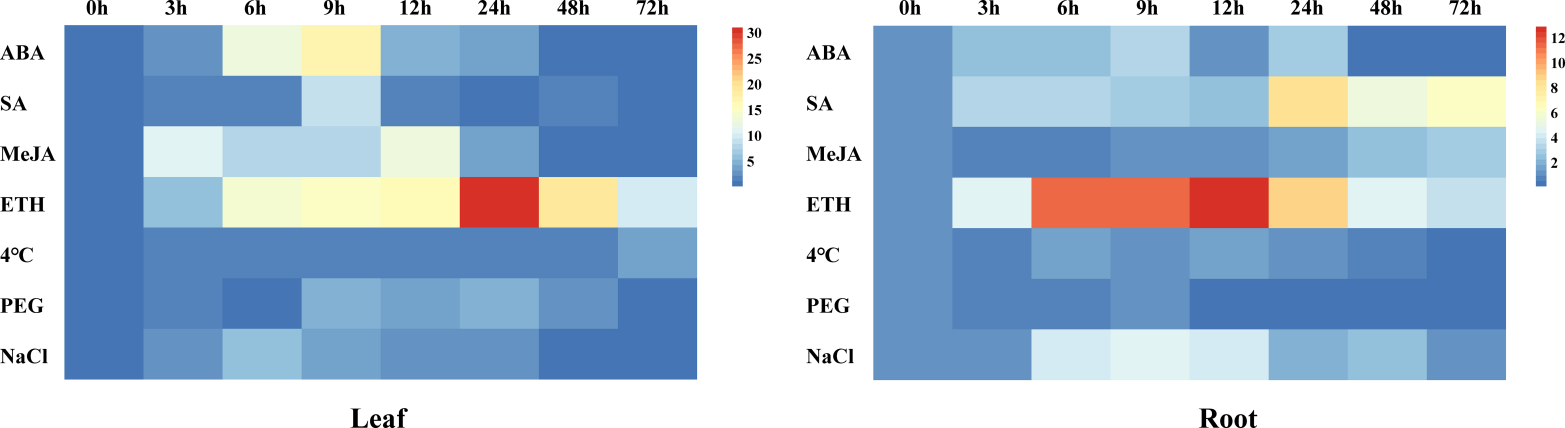
Heatmap illustrating gene expression changes of *CsSAMDC3* in cucumber leaf and root under different hormones and stress treatments.

As shown in **Figure 4**, under ABA treatment, the expression of *CsSAMDC3* in cucumber leaves and roots increased and reached the peak value at 9 h, then decreased to the average level at 48 h. Under SA treatment, the expression of *CsSAMDC3* in cucumber leaves fluctuated less compared with ABA treatment, and the peak value also appeared at 9 h, while the expression of *CsSAMDC3* in cucumber roots reached the highest at 24 h, and the response was more intense. Under MeJA treatment, the expression of *CsSAMDC3* in leaves increased at 3 h, decreased slightly, increased to the maximum at 12 h, and then decreased to the standard value. In roots, the response of *CsSAMDC3* to MeJA was not strong, but the overall trend was upward. Under ETH treatment, the expression of *CsSAMDC3* in leaves and roots was very prominent, reaching the peak at 24 h and 12 h, respectively, and the response of *CsSAMDC3* to ETH was the strongest compared with other treatment groups. For 4 °C stress and PEG simulated drought stress, the overall response of *CsSAMDC3* fluctuated less, especially in the leaves under cold stress and the roots under drought stress; The expression level has been maintained at a low level. Under salt stress, the expression levels of *CsSAMDC3* in leaves and roots reached the peak at 6 h and 9 h, respectively, and the response in roots was more robust. Compared with other abiotic stress treatments, the expression of *CsSAMDC3* reached the peak earlier under NaCl stress, indicating that *CsSAMDC3* was more sensitive to salt stress.

### Construction of tobacco overexpressing *CsSAMDC3*

**Figure 5:**
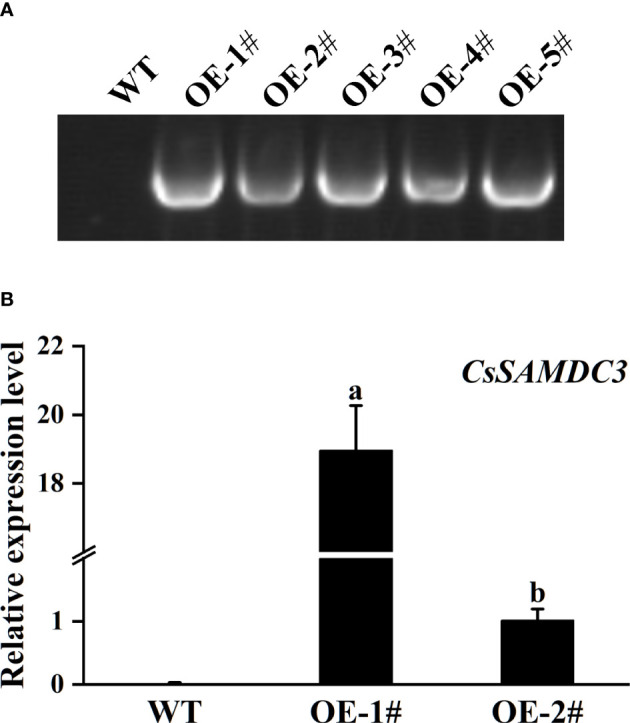
Identification of the *CsSAMDC3*-overexpressing transgenic tobacco. **(A)** Genomic DNA amplification in wild-type and overexpression transgenic plants. **(B)** qRT-PCR tested in transgenic lines and wild type. Expression level in OE-2# is taken as 1. Each value is shown as the mean ± standard error of three biological replicates. Different letters indicate significant differences at *P* < 0.05, according to Duncan’s multiple range tests.

To further study the function of *CsSAMDC3* under salt stress, the genetic transformation of tobacco was carried out by the *Agrobacterium*-mediated leaf disc method. Positive plants were screened by kanamycin, and DNA was extracted and verified by PCR with PAC019-F/PAC019-*CsSAMDC3*-R (**Supplemental Table 1**) (**Figure 5A**). The expression of *CsSAMDC3* gene in two lines with good seed quality was detected by qRT-PCR. It can be seen from **Figure 5B** that the expression level of *CsSAMDC3* in the transgenic line OE-1# was 19 times higher than that in the transgenic line OE-2#.

### Effects of overexpression of *CsSAMDC3* on biomass and photosynthetic capacity of tobacco under salt stress

**Figure 6:**
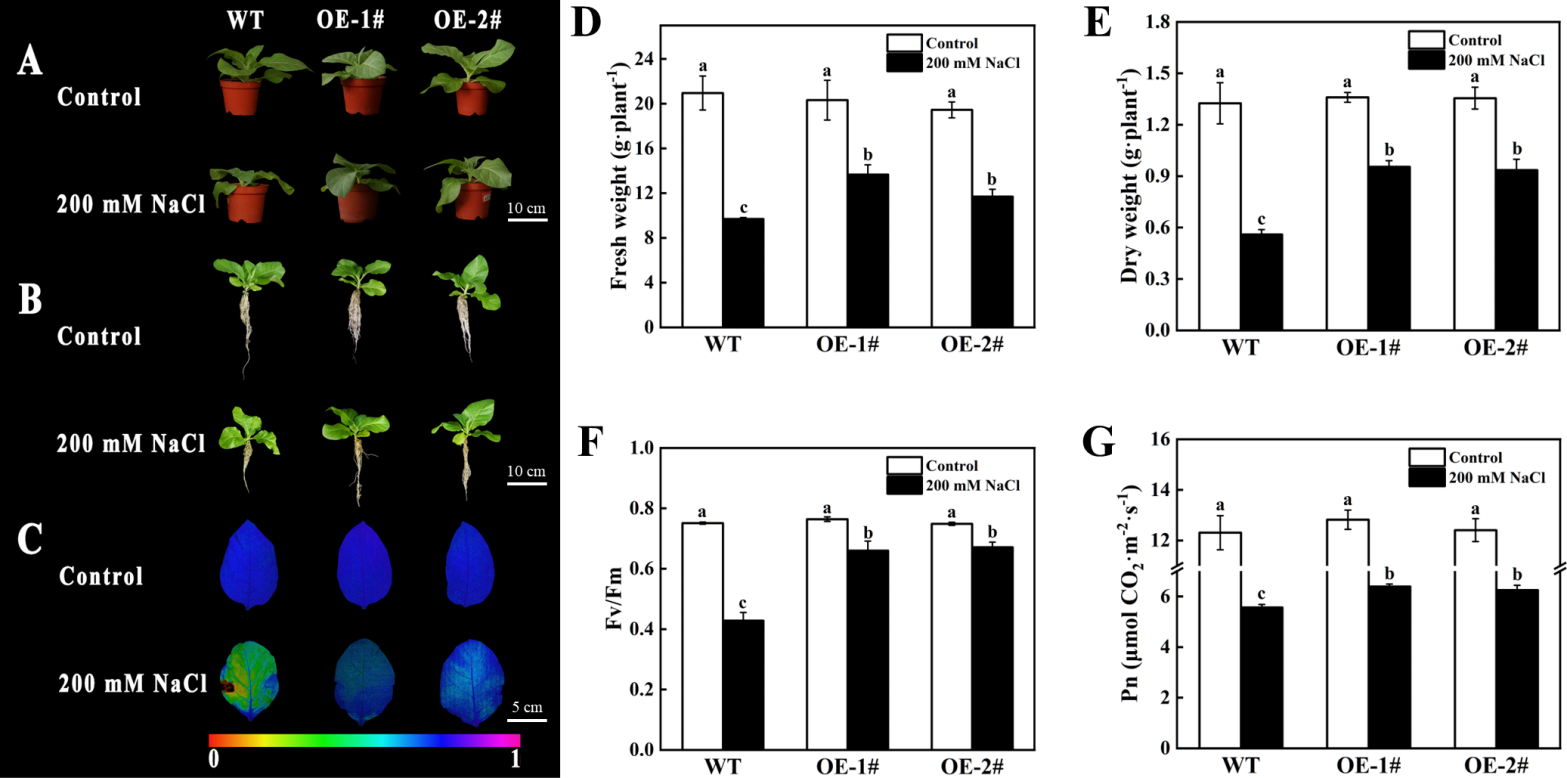
Phenotype and photosynthetic capacity analysis of the wild type and *CsSAMDC3*-overexpressing transgenic lines under salt stress. **(A, B)** Phenotype. **(C, F)** maximal photochemical efficiency. **(D)** Fresh weight. **(E)** Dry weight. **(G)** Net photosynthetic rate. Each value is shown as the mean ± standard error of three biological replicates. Different letters indicate significant differences at *P* < 0.05, according to Duncan’s multiple range tests.

As shown in **Figures 6A, B**, after 5 days of 200 mM NaCl treatment, the growth of wild-type and overexpressed *CsSAMDC3* tobacco was inhibited, showed different degrees of salt damage: compared with overexpressed plants, wild-type plants were shorter and smaller, and root growth was more significantly inhibited. Among them, the fresh and dry weight of the overexpression lines OE-1# and OE-2# decreased by 32.73%, 39.83% and 29.78%, 31.00%, respectively, compared with the control group. In contrast, the wild type decreased more significantly, 53.71% and 57.74%, respectively (**Figures 6D, E**). In addition, the Fv/Fm and Pn values of wild-type tobacco after salt treatment were significantly lower than those of *CsSAMDC3*-overexpressing tobacco, which were 36.17% and 13.00% lower, respectively (**Figures 6C, F, G**). The above results showed that the growth and ФPSII of wild-type tobacco were more severely inhibited under salt stress, and overexpression of *CsSAMDC3* alleviated the growth inhibition induced by salt stress.

### Effects of overexpression of *CsSAMDC3* on antioxidant capacity of tobacco under salt stress

**Figure 7:**
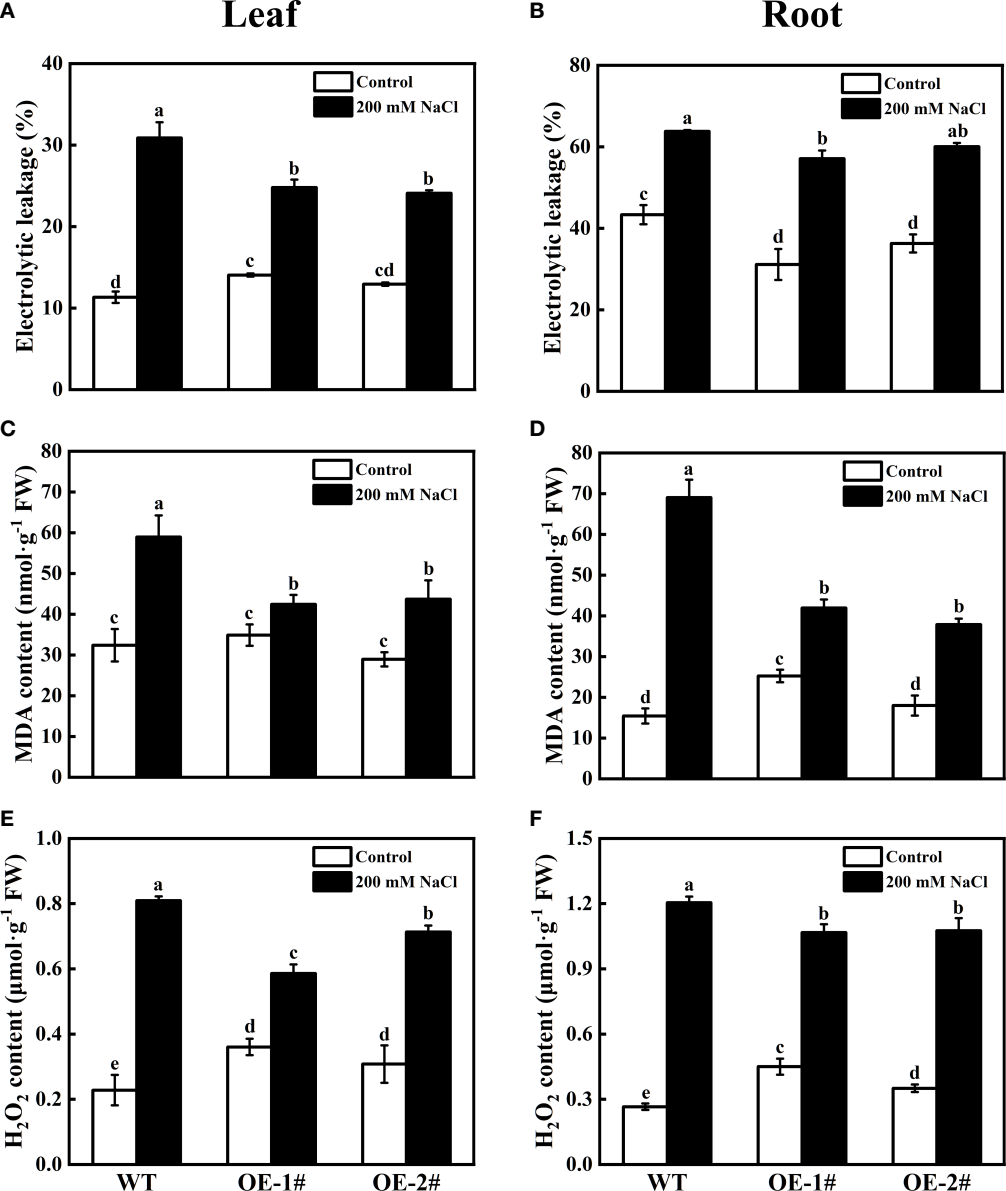
Electrolyte leakage, MDA, and H_2_O_2_ content analysis of wild type and *CsSAMDC3*-overexpressing transgenic lines under salt stress. **(A, B)** Electrolyte leakage. **(C, D)** MDA content. **(E, F)** H_2_O_2_ content. Each value is shown as the mean ± standard error of three biological replicates. Different letters indicate significant differences at *P* < 0.05, according to Duncan’s multiple range tests.

**Figure 8:**
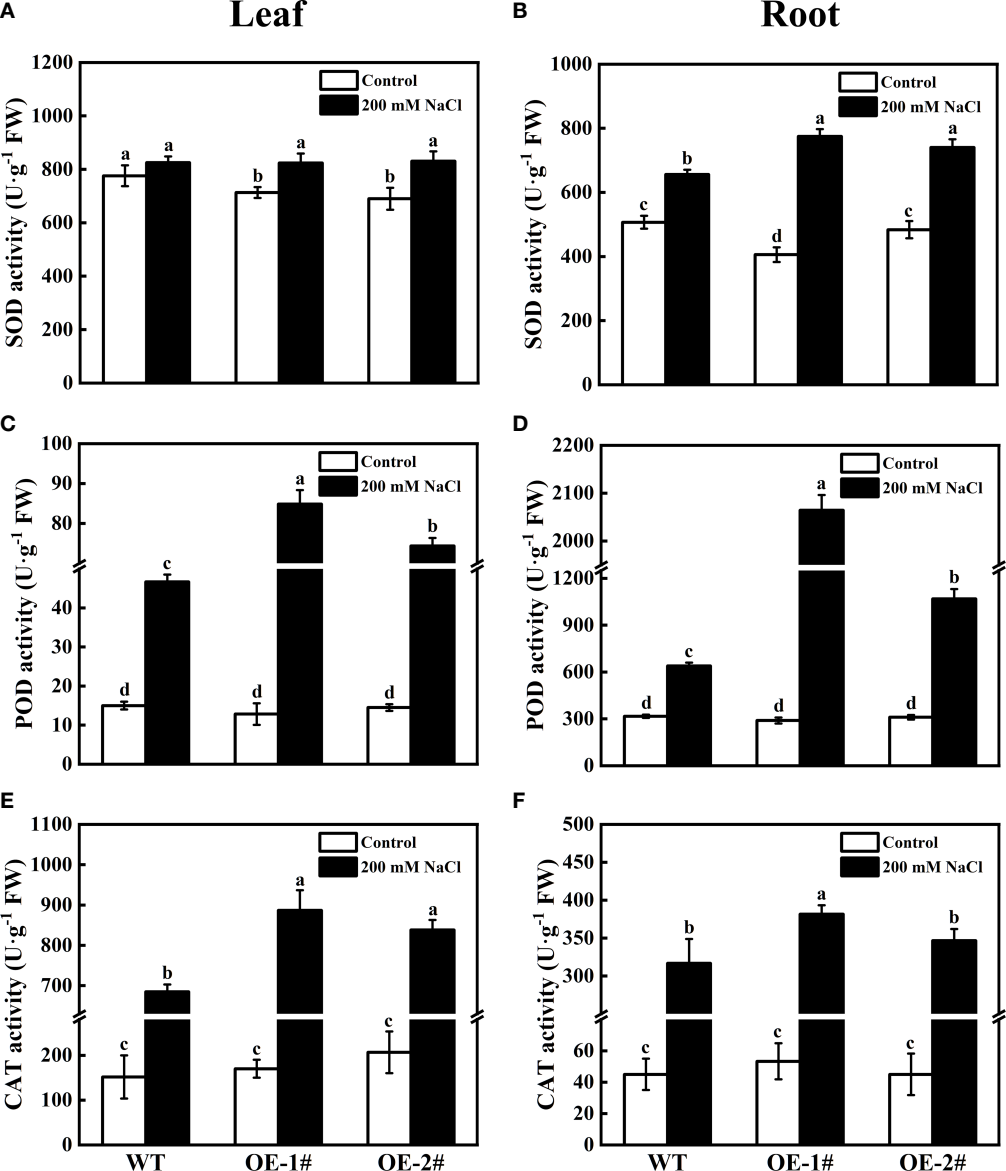
Antioxidant enzyme activity of wild-type and *CsSAMDC3*-overexpressing transgenic lines under salt stress. **(A, B)** SOD activity. **(C, D)** POD activity. **(E, F)** CAT activity. Each value is shown as the mean ± standard error of three biological replicates. Different letters indicate significant differences at *P* < 0.05, according to Duncan’s multiple range tests.

To verify the role of *CsSAMDC3* in salt stress, we further analyzed the oxidative indexes of wild-type and *CsSAMDC3*-overexpressing tobacco under salt stress. As shown in **Figure 7**, the electrical conductivity, MDA content, and H_2_O_2_ content of overexpressing plants after salt treatment were significantly lower than those of wild-type plants in both leaves and roots. Among them, the electrical conductivity of OE-1# and OE-2# decreased more in leaves, which were 19.75% and 21.99%, respectively (**Figure 7A**). MDA content decreased more in roots, 39.24% and 45.16%, respectively (**Figure 7D**). H_2_O_2_ content in leaves decreased by 27.64% and 11.91%, respectively (**Figure 7E**). In addition, we determined the activities of antioxidant enzymes in leaves and roots of wild-type and *CsSAMDC3*-overexpressing tobacco under salt stress. The results showed that for SOD, the activity of *CsSAMDC3-*overexpressing tobacco in roots was substantially higher than that of wild-type tobacco, whereas the activity in leaves was comparable to that of wild-type tobacco (**Figures 8A, B**). For POD, the activity of *CsSAMDC3-*overexpressing tobacco in roots and leaves was significantly higher than that of wild-type tobacco, and the activity of OE-1# in leaves and roots was 81.79% and 223.00% higher than that of WT, respectively (**Figures 8C, D**); For CAT, the activities of OE-1# and OE-2# in leaves were 29.44% and 22.38% higher than WT, respectively (**Figure 8E**). The activities of OE-1# and OE-2# in roots were 20.53% and 9.47% higher than WT, respectively (**Figure 8F**). Overall, overexpression of *CsSAMDC3* in tobacco can reduce MDA and H_2_O_2_ content by increasing antioxidant enzyme activity, thereby enhancing the tolerance of tobacco to salt stress.

**Figure 9:**
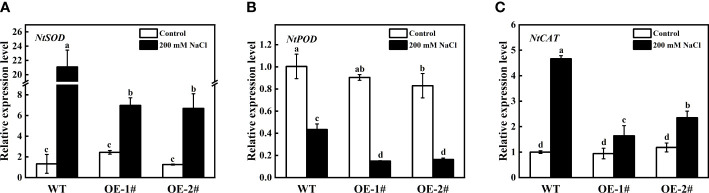
Effects of salt stress on relative gene expression of *NtSOD* **(A)**, *NtPOD* **(B)**, *NtCAT* **(C)** of wild-type and *CsSAMDC3*-overexpressing transgenic tobacco seedling roots. Each value is shown as the mean ± standard error of three biological replicates. Different letters indicate significant differences at *P* < 0.05, according to Duncan’s multiple range tests.

The expression levels of antioxidase-related coding genes (*NtSOD*, *NtPOD*, *NtCAT*) were detected. As shown in **Figure 9**, compared with WT, the expression levels of *NtSOD*, *NtPOD*, and *NtCAT* in OE-1# and OE-2# were significantly decreased under salt stress. Among them, the expression of *NtSOD* was the most obvious difference, and OE-1# and OE-2# were 66.92% and 68.26% lower than WT, respectively (**Figure 9A**). Under the control conditions, except *NtPOD*, the expression difference of other antioxidant enzyme related coding genes was not significant between transgenic tobacco and wild type.

### Effects of salt stress on polyamine metabolism in *CsSAMDC3*-overexpressing tobacco

**Figure 10:**
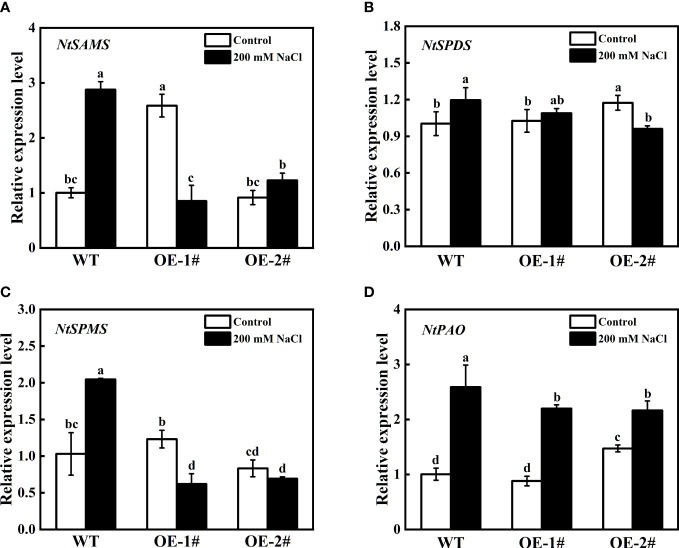
Total polyamine content of wild-type and *CsSAMDC3*-overexpressing transgenic lines under salt stress. **(A)** Put. **(B)** Spd. **(C)** Spm. **(D)** The value of (Spd + Spm)/Put. Each value is shown as the mean ± standard error of three biological replicates. Different letters indicate significant differences at *P* < 0.05, according to Duncan’s multiple range tests.

As shown in **Figure 10**, transgenic tobacco plants had lower Put content and higher Spd and Spm content compared to wild type under the control conditions. Compared with WT, the Spd and Spm content in OE-1# increased by 12.78% and 42.21% respectively, while the Put content decreased by 29.78%; the Spd and Spm content in OE-2# increased by 4.40% and 33.98% respectively, while the Put content decreased by 18.88%. However, under salt stress, the content of Put, Spd, and Spm in OE-1# decreased by 16.71%, increased by 28.81%, and increased by 45.15% compared with WT, respectively. The content of Put, Spd, and Spm in OE-2# decreased by 2.87%, increased by 21.25% and 51.68%, respectively, compared with WT. The difference between Spd and Spm content of *CsSAMDC3*-overexpressing tobacco and wild type under salt stress was further widened (**Figures 10B, C**). The values of (Spd + Spm)/Put in OE-1# and OE-2# were increased under salt stress compared with WT (**Figure 10D**). This indicates that overexpression of *CsSAMDC3* under salt stress can promote the synthesis of more Spd and Spm in transgenic tobacco.

**Figure 11:**
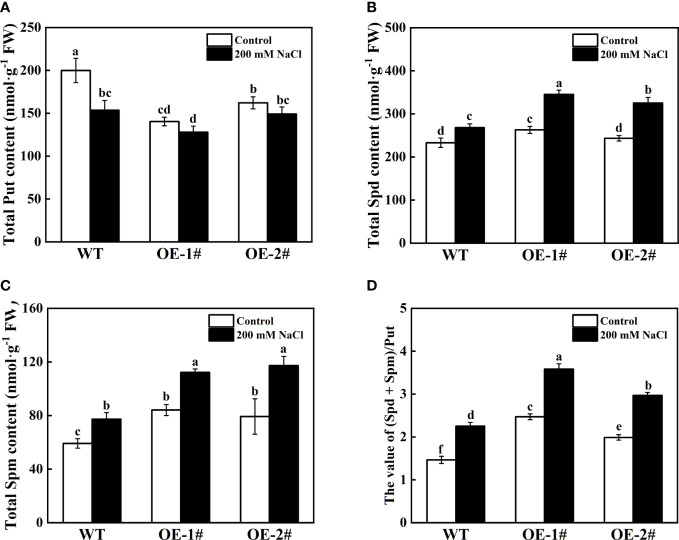
Effects of salt stress on relative gene expression of *NtSAMS* **(A)**, *NtSPDS* **(B)**, *NtSPMS* **(C)**, and *NtPAO* **(D)** of wild-type and *CsSAMDC3*-overexpressing transgenic tobacco seedling roots. Each value is shown as the mean ± standard error of three biological replicates. Different letters indicate significant differences at *P* < 0.05, according to Duncan’s multiple range tests.

As shown in **Figure 11**, under control conditions, the expression levels of *NtSAMS* and *NtSPMS* in OE-1# were significantly higher than those in WT and OE-2#, and the expression levels of *NtSPDS* and *NtPAO* in OE-2# were significantly higher than those in WT and OE-1#. However, under salt stress, compared with WT, the expression levels of PAs metabolism-related coding genes (*NtSAMS*, *NtSPDS*, *NtSPMS*, *NtPAO*) in OE-1# and OE-2# were decreased.

## Discussion

High salinity can cause osmotic and ion stress, interfering with plant growth and metabolism (Wang et al., 2022). PAs play a critical role in enhancing plant resistance. Changes in endogenous PAs content or morphology affect plant tolerance to abiotic stresses (Roy and Ghosh, 1996; Bouchereau et al., 1999; Tassoni et al., 2008; Alcázar et al., 2010). As one of the main rate-limiting enzymes in polyamine synthesis and metabolism, S-adenosylmethionine decarboxylase (SAMDC) inevitably affects the changes of endogenous PAs in plants. However, reports on cucumber SAMDC are still rare, especially at the genetic level. Therefore, it is of great significance to study the mechanism of cucumber SAMDC in salt stress from the gene perspective.

In this study, we first analyzed and identified the cucumber *SAMDCs* gene sequences by bioinformatics and studied the response of the cucumber *SAMDCs* gene to salt stress, which laid a research foundation. The cucumber *SAMDCs* gene family has four homologous genes (*CsSAMDC1-4*), and the molecular weight of the encoded protein is similar to many species, about 40 KDa (Mad Arif et al., 1994; Li and Chen, 2000; Tian et al., 2004; Zhao et al., 2017). Analysis of the full-length SAMDC protein sequences from 13 species revealed that CsSAMDC2 and CsSAMDC3 were in the same evolutionary group (**Figure 1A**), but they were located on different chromosomes (**Table 1**). Furthermore, SAMDC proteins are highly conserved in evolution and have a typical SAMDC protease domain (**Figure 1C**), which is close to the molecular weight of SAMDC proteins in many species. The response of different cucumber *SAMDCs* genes to salt stress was also different. The expression levels of *CsSAMDC2* and *CsSAMDC3* increased first and then decreased (**Figure 2**), which was consistent with the response patterns of other plant *SAMDCs* to salt stress (Peng et al., 2013; Ji et al., 2019). In addition, their expression levels peaked earlier than *CsSAMDC1* and *CsSAMDC4*, so we infer that they are more sensitive to salt stress.

Then we chose the *CsSAMDC3* gene for further study. Tissue-specific expression analysis showed that the *CsSAMDC3* gene was strongly expressed in cucumber fruits and flowers (**Figure 3A**), indicating that the expression of *CsSAMDC3* was related to flower organ development. Falasca et al. (2010) reported that the *SAMDC* gene was expressed in germinated pollen and played an essential role in pollen maturation. Subcellular localization showed that CsSAMDC3 was localized on the cell membrane and nucleus (**Figure 3B**). Through different hormone treatments, we found that *CsSAMDC3* had different degrees of response to ABA, SA, MeJA, and ETH treatments (**Figure 4**), indicating that *CsSAMDC3* may be involved in its signaling pathway. Among them, the response of *CsSAMDC3* to ETH is very intense, which indicates that excessive exogenous ETH can promote the expression of the *CsSAMDC3* gene, so that the common precursor SAM of PAs and ethylene biosynthesis pathway is more inclined to PAs synthesis and metabolism, and the accumulation of PAs will interfere with the biosynthesis of ethylene and reduce the ethylene content in plants (Bregoli et al., 2002; Madhulatha et al., 2014). In addition, through different abiotic stress treatments, we found that the response of *CsSAMDC3* to cold and drought stress was not prominent, but it was more sensitive to salt stress.

Plasma membrane damage induced by salt stress is directly related to the increase of highly toxic oxygen free radicals (Hernandez et al., 1993), resulting in MDA accumulation, lipid peroxidation, and electrolyte leakage (Lutts et al., 1996; Liang et al., 2003; Mansour, 2013). High concentration of salt will destroy plant photosynthetic system and reduce the photosynthetic rate to inhibit plant growth (Shu et al., 2013; Zahra et al., 2022). In tobacco, overexpression of *CsSAMDC3* increased the activity of antioxidant enzymes (**Figure 8**), alleviated the oxidative damage caused by salt stress (**Figure 7**), and enhanced the salt tolerance of tobacco in photosynthesis and growth (**Figure 6**). In addition, the accumulation of ROS in *CsSAMDC3*-overexpressing tobacco decreased under salt stress, which may be related to the high content of polyamines, especially Spd and Spm (**Figure 10**). Studies have shown that the ratio of (Spd + Spm)/Put in plants increases with environmental salinity (Zapata et al., 2004). Fan et al. (2013) found that high (Spd + Spm)/Put ratio and Spm accumulation were beneficial to improve the salt tolerance of cucumber seedlings. In this study, overexpression of *CsSAMDC3* resulted in the accumulation of Spd and Spm and the reduction t of Put in tobacco (**Figure 10**), thereby improving the salt tolerance of tobacco. Jia et al. (2021), who overexpressed *TrSAMDC1* in *Arabidopsis thaliana*, found that overexpression of *TrSAMDC1* can enhance the tolerance of *Arabidopsis thaliana* to salt and drought stress by increasing endogenous PAs levels and antioxidant enzyme activity. Similarly, Wi et al. (2014) obtained a similar conclusion by overexpressing *CaSAMDC* in *Arabidopsis thaliana*. Interestingly, we determined the expression levels of antioxidant enzymes and polyamine metabolism-related genes in tobacco and found that they were negatively correlated with antioxidant enzyme activity or polyamine accumulation (**Figures 9**, **11**). This suggests that enhanced antioxidant enzyme activity or polyamine accumulation may negatively regulate the expression of related genes, thereby maintaining a relatively stable dynamic balance in plants (Xu et al., 1999; Michelet et al., 2011).

## Conclusion

In summary, we identified four *SAMDC* genes (*CsSAMDC1-4*) in cucumber and divided them into three groups. The CsSAMDC2 and CsSAMDC3 encoding genes in the same group 1 showed similar salt stress response patterns. *CsSAMDC3* is highly expressed in flowers and fruits, indicating that it plays a vital role in reproductive growth.Overexpression of *CsSAMDC3* in tobacco confirmed that *CsSAMDC3* could increase Spd and Spm content, increase antioxidant enzyme activity, enhance plant salt tolerance by scavenging reactive oxygen species, and is a candidate gene for improving plant salt tolerance. The results of this study laid a foundation for further study on the mechanism of polyamines regulating the salt tolerance of cucumber at the molecular level. In the future, more exploration and evidence are needed to clarify the role and relationship of *CsSAMDCs* in plant stress resistance.

## Data availability statement

The datasets presented in this study can be found in online repositories. The names of the repository/repositories and accession number(s) can be found in the article/**Supplementary material**.

## Author contributions

SS designed the experimental research content. MZ performed the experiments and wrote the manuscript. GC provided some technical assistance for experiments about the genetic transformation of tobacco. JQW, JW, YW and SG modified the manuscript. All authors contributed to the article and approved the submitted version.
