# Supplementary material for: Identification of cucumber S-adenosylmethionine decarboxylase genes and functional analysis of CsSAMDC3 in salt tolerance
Source: Front Plant Sci. 2023 Apr 21;14:1076153. doi: 10.3389/fpls.2023.1076153 (PMC10162440; doi:10.3389/fpls.2023.1076153)
Supplement: Supplementary file 1 [file DataSheet_1.docx]

**Supplementary data**

**Supplemental Figure 1.** Subcellular localization prediction of CsSAMDC3.

**
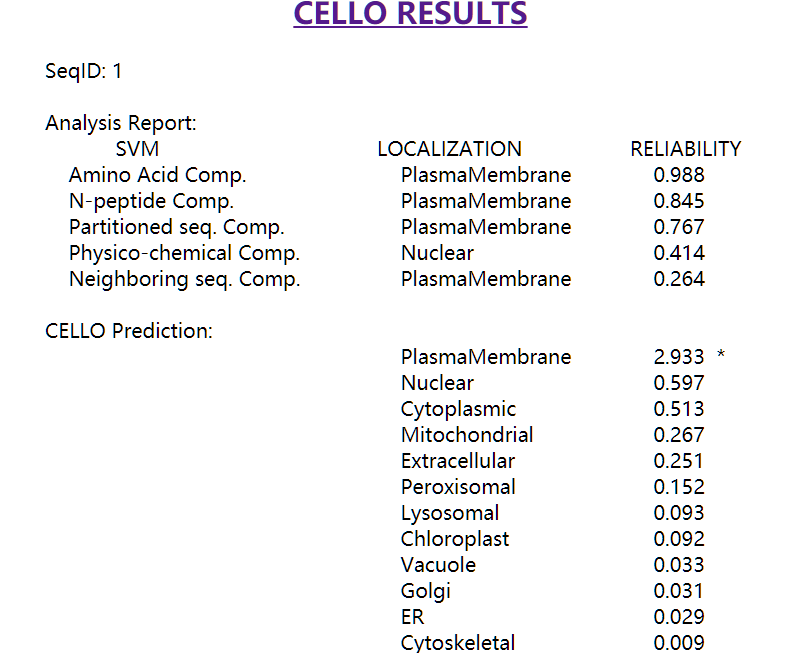
**

**Supplemental Figure 2.** Gene expression changes of *CsSAMDC3* in cucumber leaf and root under different hormones and stress treatments.


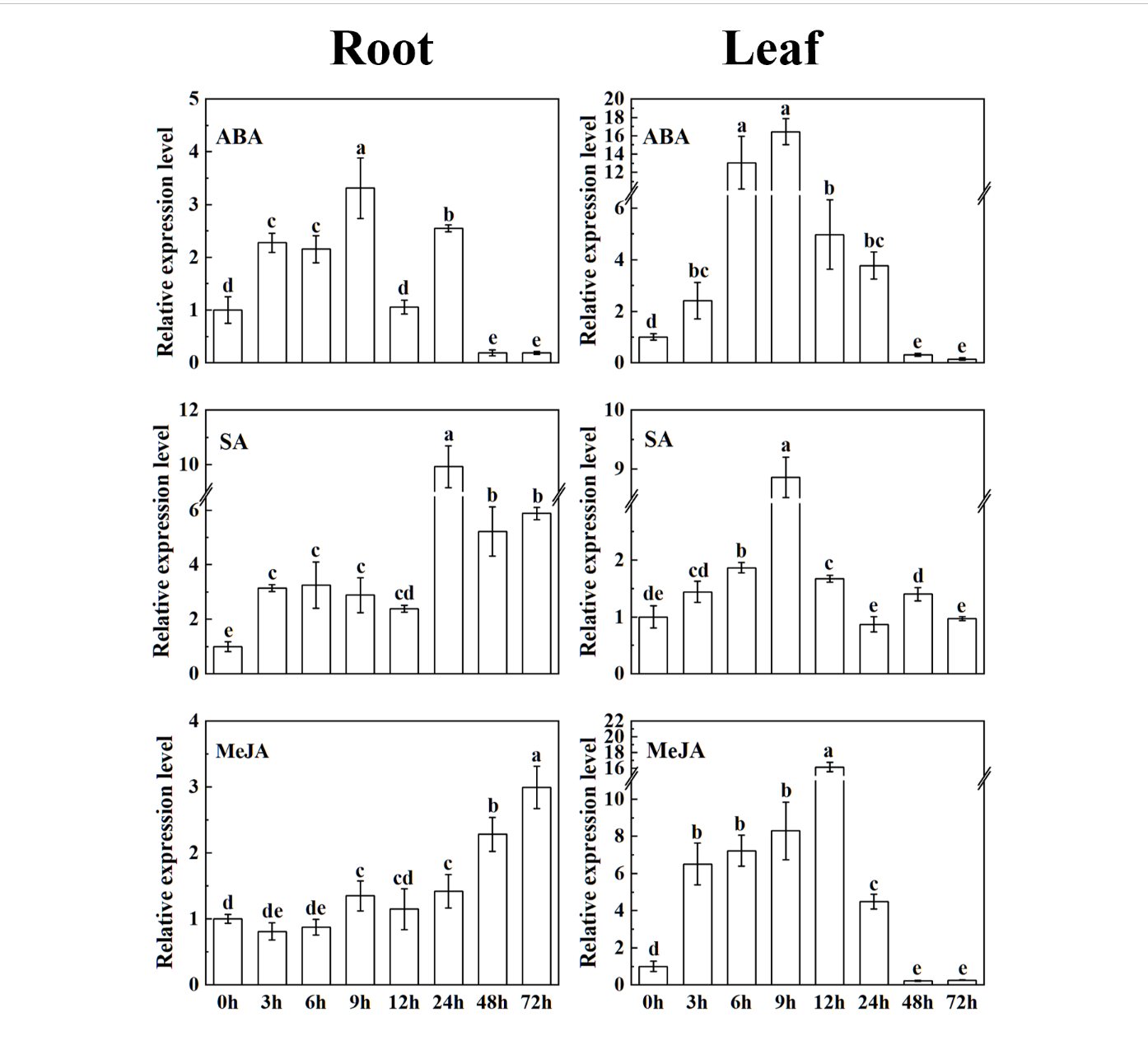
**
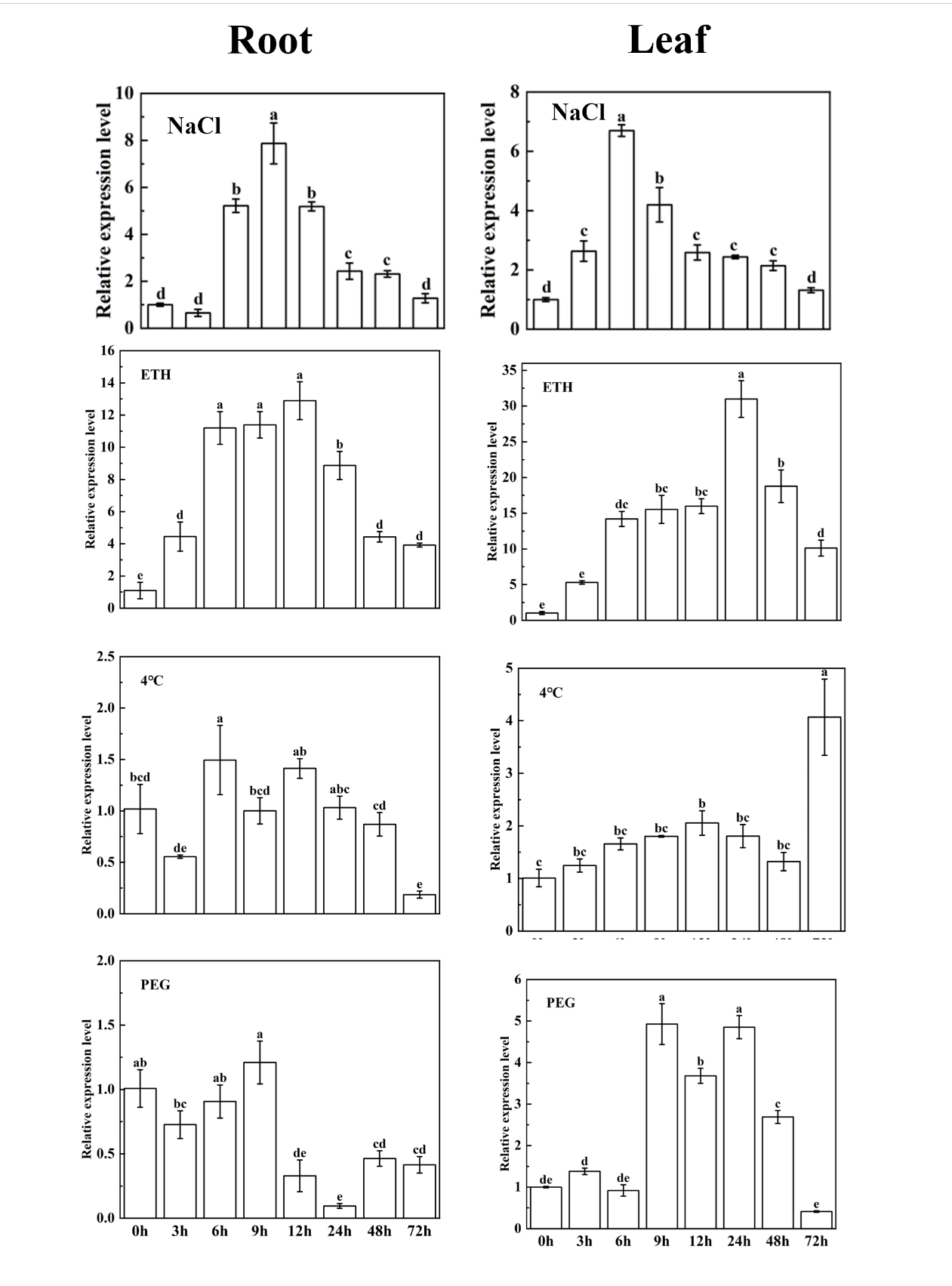
**

**Supplemental Table 1.** Primers used for this study.

| Primer name | Primer sequence (5'-3') | Function |
| --- | --- | --- |
| PAC019-*CsSAMDC3*-F  PAC019-*CsSAMDC3*-R  PAC019-F  Cs*ACTIN*-F  Cs*ACTIN*-R  q*CsSAMDC1*-F  q*CsSAMDC1*-R  q*CsSAMDC2*-F  q*CsSAMDC2*-R  q*CsSAMDC3*-F  q*CsSAMDC3*-R  q*CsSAMDC4*-F  q*CsSAMDC4*-R  q*NtACTIN*-F  q*NtACTIN*-R  q*NtPAO*-F  q*NtPAO*-R  q*NtSPMS*-F  q*NtSPMS*-R  q*NtSPDS*-F  q*NtSPDS*-R  q*NtPOD*-F  q*NtPOD*-R  q*NtSOD*-F  q*NtSOD*-R  q*NtCAT*-F  q*NtCAT*-R | gagaacacgggggactctagaATGGCGGAGTCTGGTTTTGA | Overexpression construct of *CsSAMDC3* |
|  | gcccttgctcaccatggtaccTTTCCGGCGAGCCGTGAA |  |
|  | CAATCCCACTATCCTTCGC | Transgenic identification |
|  | CAGGAATCCACGAAACTACT  AGACCCTCCAATCCAAACAC  GTTGATTGTGAGGGTGCT  TCTGGTAGATGACGGTTC  CCTCCCTAACCACCTCTG  AAATACCTGGACGGCTCT  AAAGCCTCCGTTATCCCT  GCGTAACTAAACCCATCCT  TCGCAACTGGCACATCTA  CCATCTCCTGCTTCACAA | qRT-PCR analysis for gene expression |
|  | CCTGAGGTCCTTTTCCAACCA  GGATTCCGGCAGCTTCCATT  GTCGCTGCTCTGTCGTCATAGTC  CCTCCTTCCTTATCCTGCCACCT  CGACTGAGGGACCACCTGTTGA  ACAAAGGACGGCAAGGCAAAGG  GGAAGCAGCCAACCACAACAAC  CAGAGAACCAGCCAGGAAGAACA  ATGTCCTGACCCAATCCC  ATCTCACCCTTTGTTCCA  AGCTACATGACGCCATTTCC  CCCTGTAAAGCAGCACCTTC  AGGTACCGCTCATTCACACC  AAGCAAGCTTTTGACCCAGA |  |

**Supplemental Table 2.** The SAMDC proteins used in this study.

| Species | Protein name | Accession numbers |
| --- | --- | --- |
| *Solanum lycopersicum*  *Solanum lycopersicum*  *Cucumis sativus*  *Cucumis sativus*  *Cucumis sativus*  *Cucumis sativus*  *Zea mays*  *Zea mays*  *Zea mays*  *Zea mays*  *Brassica napus*  *Brassica napus*  *Brassica napus*  *Brassica napus*  *Capsicum annuum*  *Capsicum annuum*  *Arachis hypogaea*  *Arachis hypogaea*  *Arachis hypogaea*  *Arachis hypogaea*  *Arachis hypogaea*  *Vitis vinifera*  *Vitis vinifera*  *Vitis vinifera*  *Oryza sativa*  *Oryza sativa*  *Oryza sativa*  *Oryza sativa*  *Glycine max*  *Glycine max*  *Glycine max*  *Glycine max*  *Arabidopsis thaliana*  *Arabidopsis thaliana*  *Arabidopsis thaliana*  *Nicotiana tabacum*  *Nicotiana tabacum*  *Nicotiana tabacum*  *Nicotiana tabacum*  *Nicotiana tabacum*  *Nicotiana tabacum*  *Malus domestica*  *Malus domestica*  *Malus domestica*  *Cucurbita moschata*  *Cucurbita moschata*  *Cucurbita moschata*  *Cucurbita moschata* | SlSAMDC1  SlSAMDC2  CsSAMDC3  CsSAMDC2  CsSAMDC1  CsSAMDC4  ZmCsAMDC1  ZmCsAMDC2  ZmCsAMDC3  ZmCsAMDC4  BnSAMDC1  BnSAMDC2  BnSAMDC3  BnSAMDC4  CaSAMDC4  CaSAMDC1  AhSAMDC4  AhSAMDC3  AhSAMDC5  AhSAMDC2  AhSAMDC1  VvSAMDC4  VvSAMDC2  VvSAMDC3  OsSAMDC1  OsSAMDC2  OsSAMDC3  OsSAMDC4  GmSAMDC1  GmSAMDC2  GmSAMDC3  GmSAMDC4  AtSAMDC1  AtSAMDC3  AtSAMDC4  NtSAMDC1  NtSAMDC2  NtSAMDC4  NtSAMDC5  NtSAMDC6  NtSAMDC3  MdSAMDC1  MdSAMDC2  MdSAMDC4  CmSAMDC4  CmSAMDC1  CmSAMDC2  CmSAMDC3 | NP_001307075.1  NP_001307257.1  XP_004134166.1  XP_004145040.2  XP_031738092.1  XP_031737140.1  NP_001148939.1  NP_001149694.1  NP_001105713.1  NP_001149266.1  XP_013738638.1  XP_013740200.1  XP_013735693.1  XP_013680534.1  XP_016557034.1  XP_016577115.1  XP_025611767.1  XP_025693772.1  XP_025694029.1  XP_025654995.1  XP_025696905.1  XP_002277316.1  XP_010656642.2  XP_002282708.1  XP_025876108.1  XP_025881009.1  XP_015635570.1  XP_015638505.1  XP_006602989.1  XP_003550675.1  XP_003550518.1  XP_003528663.1  NP_001154585.1  NP_001189972.1  NP_001031888.1  NP_001312627.2  XP_016477309.1  XP_016470301.1  XP_016459994.1  XP_016499007.1  XP_016472420.1  NP_001315970.2  NP_001280825.1  XP_008390797.2  XP_022959322.1  XP_022948952.1  XP_022946262.1  XP_022955404.1 |

**Supplemental Table 3.** Sequences and lengths of motifs among SAMDC proteins of different plant species.

| Motif | Conserved amino acid sequences | Width |
| --- | --- | --- |
| 1  2  3  4  5  6  7  8  9  10  11  12  13  14  15  16 | MTELSGIRKILPNSEICDFEFDPCGYSMNGIE  CTIVSSLSNDDFDSYVLSESSLFVYPYKIIIKTCGTTKLLLSIPPJLKLA  LSLTVKSVRYTRGSFIFPGAQPFPHRSFSEEVAVLBGYFGK  GDAYSTIHVTPEDGFSYASFEAVG  PVYTLEMCMTGLDREKASVFFKTPGDGAA  AIGFEGFEKRLEJTFF  EPPVFADPEGLGLRALSKAQJDEILDPAE  QLVKRVLQCFRPAEFSVAVTC  MGSADPSQKWHVYSA  EELPGGGSVVYQTFT  VWTRVAGAVEPLGLKCRSCAV  CGSPRSVLKCC  LGLDVKGYECLEQVL  LGSGSAAY  FGGRGHAGTWA  YDFKTVBL | 32  50  41  24  29  16  29  21  15  15  21  11  15  8  11  8 |
